# Corticotrophin-Releasing Factor Modulates the Facial Stimulation-Evoked Molecular Layer Interneuron-Purkinje Cell Synaptic Transmission *in vivo* in Mice

**DOI:** 10.3389/fncel.2020.563428

**Published:** 2020-11-26

**Authors:** Wen-Yuan Wu, Yang Liu, Mao-Cheng Wu, Hong-Wei Wang, Chun-Ping Chu, Hua Jin, Yu-Zi Li, De-Lai Qiu

**Affiliations:** ^1^Department of Physiology and Pathophysiology, College of Medicine, Yanbian University, Yanji, China; ^2^Brain Science Research Center, Yanbian University, Yanji, China; ^3^Department of Urology, Affiliated Hospital of Yanbian University, Yanji, China; ^4^Department of Osteology, Affiliated Hospital of Yanbian University, Yanji, China; ^5^Department of Cardiology, Affiliated Zhongshan Hospital of Dalian University, Dalian, China; ^6^Department of Nephrology, Affiliated Hospital of Yanbian University, Yanji, China; ^7^Department of Cardiology, Affiliated Hospital of Yanbian University, Yanji, China

**Keywords:** corticotropin-releasing factor, mouse cerebellar cortex, sensory stimulation, molecular layer interneuron, Purkinje cell, *in vivo* cell-attached recording, neurobiotin juxtacellular labeling

## Abstract

Corticotropin-releasing factor (CRF) is an important neuromodulator in central nervous system that modulates neuronal activity via its receptors during stress responses. In cerebellar cortex, CRF modulates the simple spike (SS) firing activity of Purkinje cells (PCs) has been previously demonstrated, whereas the effect of CRF on the molecular layer interneuron (MLI)–PC synaptic transmission is still unknown. In this study, we examined the effect of CRF on the facial stimulation–evoked cerebellar cortical MLI-PC synaptic transmission in urethane-anesthetized mice by *in vivo* cell-attached recording, neurobiotin juxtacellular labeling, immunohistochemistry techniques, and pharmacological method. Cell-attached recordings from cerebellar PCs showed that air-puff stimulation of ipsilateral whisker pad evoked a sequence of tiny parallel fiber volley (N1) followed by MLI-PC synaptic transmission (P1). Microapplication of CRF in cerebellar cortical molecular layer induced increases in amplitude of P1 and pause of SS firing. The CRF decreases in amplitude of P1 waveform were in a dose-dependent manner with the EC_50_ of 241 nM. The effects of CRF on amplitude of P1 and pause of SS firing were abolished by either a non-selective CRF receptor antagonist, α-helical CRF-(9-14), or a selective CRF-R1 antagonist, BMS-763534 (BMS, 200 nM), but were not prevented by a selective CRF-R2 antagonist, antisauvagine-30 (200 nM). Notably, application CRF not only induced a significant increase in spontaneous spike firing rate, but also produced a significant increase in the number of the facial stimulation–evoked action potential in MLIs. The effect of CRF on the activity of MLIs was blocked by the selective CRF-R1 antagonist, and the MLIs expressed the CRF-R1 imunoreactivity. These results indicate that CRF increases excitability of MLIs via CRF-R1, resulting in an enhancement of the facial stimulation–evoked MLI-PC synaptic transmission *in vivo* in mice.

## Introduction

Corticotropin-releasing factor (CRF) is synthesized and secreted in many regions of the central nervous system and is distributed in the hypothalamus, cerebral cortex, amygdala, cerebellum, and spinal cord (Palkovits et al., 1987; Barmack and Young, 1990; Luo et al., 1994; Tian and Bishop, 2003; Ezra-Nevo et al., 2018b). In the mammalian brain, CRF is released following stress and subsequently stimulates the release of adrenocorticotropic hormone from the anterior pituitary, which has a critical role in coordinating the physiological and behavioral responses to stressors (Vale et al., 1981; Antoni, 1986; Luo et al., 1994; Hauger et al., 2009).

Two types of CRF receptors have been identified as CRF-R1 and CRF-R2 (Chen et al., 1993). CRF binds to CRF-R1 with high affinity, but has low affinity for CRF-R2 (Dautzenberg and Hauger, 2002; Hauger et al., 2003). Immunohistochemical studies have shown that both CRF-R1 and CRF-R2 were expressed in the adult rodent cerebellum (Bishop, 1990; Bishop et al., 2000; Lee et al., 2004). CRF-R1 is expressed throughout all lobules of the cerebellar cortex, including the primary dendrites and somas of Purkinje cells (PCs), molecular layer interneurons (MLIs), Golgi cells, Bergmann glial cells, and granular cells (Tian et al., 2008; Tao et al., 2009). The labeling of CRF-R2 has been found in the molecular layer, such as parallel fibers and their terminals (Tian et al., 2006). Both CRF-R1 and CRF-R2 were expressed in climbing fibers of the adult rat cerebellum (Swinny et al., 2003). Physiological studies demonstrated CRF binding to CRF receptors, consequently modulating neuronal spontaneous spike firing activity in cerebellar cortex (Fox and Gruol, 1993; Gunn et al., 2017; Prouty et al., 2017). It has been demonstrated that CRF increased the spontaneous firing rate of cerebellar PCs via CRF-R2 (Bishop et al., 2006; Tao et al., 2009), as well as by modulating sodium and potassium and hyperpolarizing activated cationic current currents in cerebellar slices (Libster et al., 2015). Our previous results demonstrated that CRF acted on presynaptic CRF-R2 of cerebellar PCs, resulting in an increase in glutamate release via PKA pathway, which contributed to modulation of the cerebellar PCs outputs *in vivo* in mice (Wang et al., 2018). Moreover, the release of CRF from climbing fibers can be reliably induced by direct electrical or chemical stimulation of the inferior olive, as well as by stimulation of specific sensory afferents (Palkovits et al., 1987; Barmack and Young, 1990; Tian and Bishop, 2003), and the reduction in CRF levels of the inferior olive nucleus is sufficient to induce motor deficiency under challenging conditions, irrespective of basal locomotion or anxiety-like behavior (Ezra-Nevo et al., 2018b). Moreover, CRFergic fibers project to granular layer as mossy fibers to regulate their synaptic transmission and plasticity (Chen et al., 2000; Refojo et al., 2011; K``uhne et al., 2012), and it acts as critical roles in regulating particular forms of cerebellar learning both at the cellular and behavioral levels, but without an effect on baseline motor skills (Ezra-Nevo et al., 2018a).

Collectively, CRF affects neuronal excitability by modulating neuronal membrane properties, and forms of synaptic transmission have been well studied, but the mechanisms of CRF modulating the sensory stimulation–evoked MLI-PC synaptic transmission in living animals remain unclear. We here studied the effect of CRF on the sensory stimulation–evoked MLI-PC synaptic transmission in urethane-anesthetized mice by *in vivo* cell-attached recording with histochemistry, immunohistochemistry techniques, and pharmacological method.

## Materials and Methods

### Anesthesia and Surgical Procedures

The anesthesia and surgical procedures have been described previously (Chu et al., 2011). In brief, the experimental procedures were approved by the Animal Care and Use Committee of Yanbian University and were in accordance with the animal welfare guidelines of the United States National Institutes of Health. The permit number is SYXK (Ji) 2011-006. ICR mice were bought from the experiment center of Jilin University and housed under a 12-h light–12-h dark cycle with free access to food and water. Either male (*n* = 37) or female (*n* = 33) adult (6–8-week-old) mice were anesthetized with urethane (1.3 g/kg body weight i.p.). A watertight chamber was created, and a 1–1.5 mm craniotomy was drilled to expose the cerebellar surface corresponding to Crus II. The brain surface was constantly superfused with oxygenated artificial cerebrospinal fluid (ACSF: 125 mM NaCl, 3 mM KCl, 1 mM MgSO4, 2 mM CaCl_2_, 1 mM NaH_2_PO_4_, 25 mM NaHCO_3_, and 10 mM D-glucose) with a peristaltic pump (Gilson Minipuls 3; Villiers-Le-Bel, France) at 0.5 mL/min. Rectal temperature was monitored and maintained at 37.0°C ± 0.2°C using body temperature equipment.

### Electrophysiological Recording and Facial Stimulation

Cell-attached recordings from cerebellar PCs and MLIs were performed with an Axopatch-200B amplifier (Molecular Devices, Foster City, CA, United States). The signal of PC spontaneous activity was acquired through a Digidata 1,440 series analog-to-digital interface on a personal computer using Clampex 10.3 software. Patch pipettes were made with a puller (PB-10; Narishige, Tokyo, Japan) from thick-wall borosilicate glass (GD-1.5; Narishige). Recording electrodes were filled with ACSF, with resistances of 3–5 MΩ. The cell-attached recordings from PCs were performed at depths of 150–200 μm under the pia mater membrane and were identified by regular spontaneous simple spikes (SSs) accompanied with irregular complex spikes (CSs). MLIs were first identified by irregular spike firing and the depth of their location in the molecular layer and were further confirmed by the neurobiotin juxtacellular labeling method (Holtzman et al., 2006; Liu et al., 2014).

**FIGURE 1:**
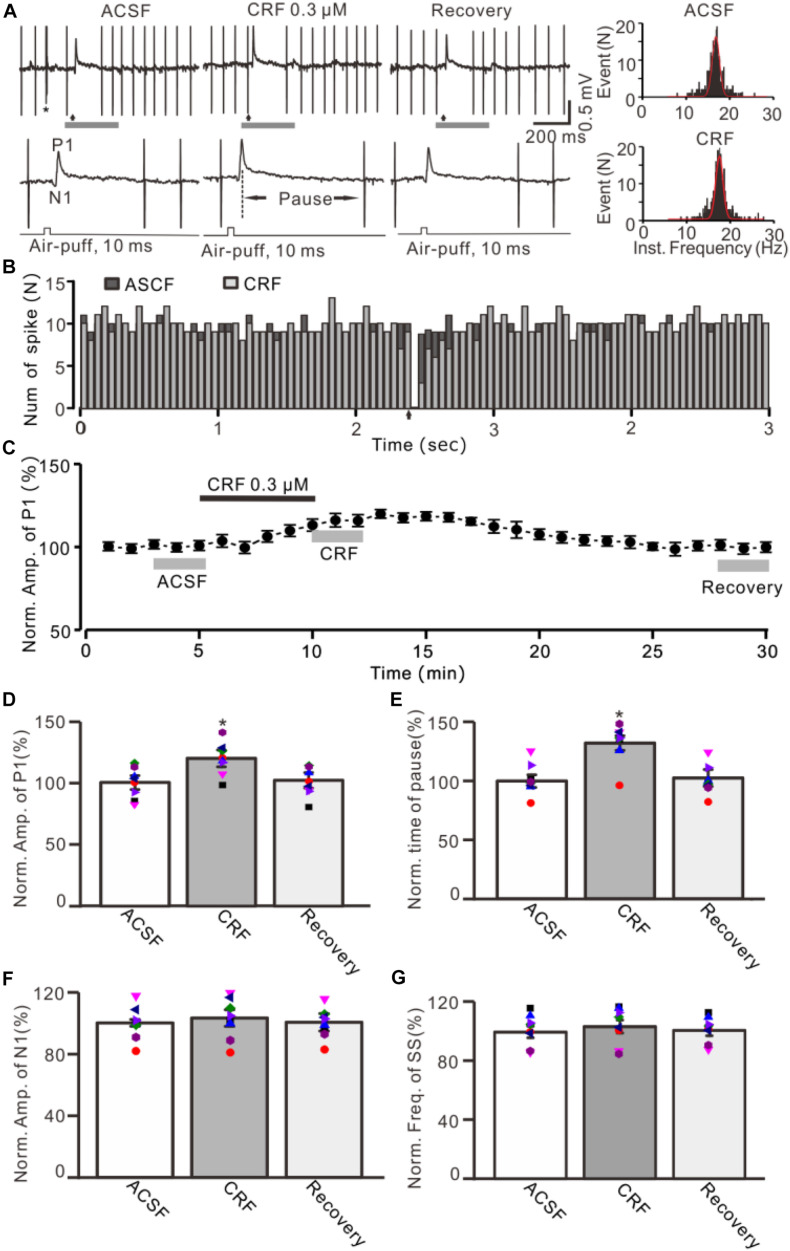
Effect of CRF on the facial stimulation–evoked MLI-PC synaptic transmission *in vivo* in mice. **(A)** Upper: Representative cell-attached recording traces showing air-puff stimulation (10 ms, 60 psi; arrows)–evoked responses in a cerebellar PC during application of ACSF, CRF (0.3 μM), and recovery (washout). Lower: Enlarged traces of the air-puff stimulation-evoked response of upper panel (gray bars). **(B)** Perievent histograms showing the simple spike discharge of a PC [shown in panel **(A)**] in the presence of ACSF (black) and CRF (gray) (arrow denotes air-puff stimulation). **(C)** Summary of data (*n* = 8) showing the time course of normalized amplitude of P1. **(D)** Summary of data (*n* = 8) shows the normalized amplitude of P1 during application of ACSF, CRF, and recovery [indicated by gray bars in panel **(C)**]. **(E)** Summary of data (*n* = 8) shows the normalized pause of simple spike firing during application of ACSF, CRF, and recovery. **(F)** Pooled data (*n* = 8) show the normalized amplitude of N1 during application of ACSF, CRF, and recovery. **(G)** Bar graph with individual data showing the mean frequency of simple spike firing during application of ACSF, CRF, and recovery. Error bars indicate SEM. *P < 0.05 vs. control.

Facial stimulation was performed by air-puff (10 ms, 60 psi) of the ipsilateral whisker pad through a 12-gauge stainless-steel tube connected with a pressurized injection system (Picospritzer^®^ III; Parker Hannifin Co., Pine Brook, NJ, United States). The air-puff stimuli were controlled by a personal computer and were synchronized with the electrophysiological recordings and delivered at 0.033 Hz via a Master 8 controller (A.M.P.I., Jerusalem, Israel) and Clampex 10.3 software. The duration of each sweep was 25 s, and the intersweep interval was 30 s. After baseline recording (10 sweeps, 5 min), CRF receptors antagonists and/or CRF were applied (Figures 1C, 3B, 4B, 5B). The durations for calculation of the mean values under control (ACSF), CRF, and recovery were indicated in the figures. The facial stimulation–evoked MLI–PC synaptic response has been demonstrated in our previous studies (Supplementary Material; Chu et al., 2011; Bing et al., 2015), which was expressed a sequence of negative components (N1) followed by a positive component (P1) accompanied with a pause of SS firing (Figure 1A).

### Histochemistry for Neurobiotin to Identify MLIs

Juxtacellular stimulation of MLIs was performed by the ejection from the micropipette using a stimulus train of positive current pulses (1–2 nA, 250 ms, 4 Hz) for 1–3 min (for approximately 40 min per experiment) (Liu et al., 2014). The whole brain was then removed and fixed in 4% paraformaldehyde (PFA) in 0.1 phosphate-buffered saline (PBS; pH 7.4) at 4°C for 24 h. Slices were cut (100 μm thick) in the sagittal plane using a vibratome (WPI, Worcester, MA, United States) and washed with PBS. The tissue was incubated overnight with an avidin-biotinylated peroxidase complex (ABC Elite kit; Vector Laboratories, Burlingame, CA, United States) at room temperature. The histochemical reaction is catalyzed by the Horseradish peroxidase (HRP) enzyme, and neurobiotin binding was detected by 3,3′-diaminobenzidine tetrahydrochloride histochemistry. Labeled neurons were visualized under microscopy (Leica DM5500; Leica, Germany) and photographed with a CCD camera (Leica).

### Immunohistochemistry and Imaging

Mice (*n* = 3) were deeply anesthetized with an intraperitoneal injection of 7% chloral hydrate (5 mL/kg) and then transcardially perfused by 300 mL cold phosphate buffer (PBS) at pH 7.4, followed by 300 mL 4% PFA (Sinopharm Chemical Reagent Co., China) PBS solution. Brain was postfixed in 4% PFA for 48 h at 4°C and washed with PBS five times at 10-min intervals. The cerebellum was separated from the brain with a razor blade. The cerebellum was exposed to 10% sucrose, 20% sucrose, and 30% sucrose in PBS for more than 6 h. After embedding in Tissue-Tek O.C.T. Compound (Beijing Zhong Shan Jin Qiao Biotechnology Co., China), the cerebellum was quickly frozen in −80°C refrigerator for 2 h. Then cerebellum was sectioned into 8-μm slices in the sagittal plane using a freezing microtome (CM1900, Leica, Germany). The sections were attached to microscope slides. Sections were rewarming at 25°C for 30 min and then fixed with 4°C precooled acetone for 20 min. Slices were stored at 4°C for immunohistochemical experiments.

Microscope slides were permeabilized with 0.3% Triton X-100 in PBS and then were blocked (10% donkey serum in PBS) and incubated in a primary antibody (rabbit anti-CRHR1, 1:300; Sigma–Aldrich), followed by Alexa Fluor 555 donkey anti-rabbit (Life Tech, 1:1,000) and 4′,6-diamidino-2-phenylindole (DAPI, 1:1,000). The incubation time for the primary antibody was overnight at 4°C. For the secondary antibody and DAPI, the incubation time was 2 h at room temperature. Microscope slides with slices were then washed three times in PBS covered with a coverslip and sealed with nail polish. Fluorescence images were acquired by confocal laser-scanning microscope (Nikon C2, Tokyo, Japan) (Mi et al., 2018). A large region image including the desired region (Crus II) was obtained on Nikon C2 laser confocal system with 10× objective.

### Chemicals

The reagents included human/rat CRF (Peptide Institute Inc., Japan); α-helical CRF-(9-14), BMS-763534, 5-chloro-1-[(1*S*)-1-cyclopropyl-2-methoxyethyl]-3-{[6- (difluoromethoxy)-2,5-dimethyl-3-pyridinyl]amino}-2(1*H*)-pyrazinone, and antisauvagine-30 were purchased from Sigma–Aldrich (Shanghai, China). The stock solutions of BMS-763534 were dissolved in dimethyl sulfoxide. CRF was dissolved in ACSF and micro applied onto the molecular layer above the recorded PCs or MLIs at 0.1 μL/s for 100 s by a micropump (KDS-210, KD Scientific, Holliston, MA, United States). The other drugs were finally dissolved in ACSF, and bath applied directly onto the cerebellar surface by a peristaltic pump (Gilson Minipulse 3; Villiers-Le-Bel, France) at 0.5 mL/min. After a stable cell attached was configured, the baseline was recorded for 100 s, then perfusion of chemicals was done.

### Statistical Analysis

Electrophysiological data were analyzed with Clampfit 10.4 (Molecular Devices, Foster City, CA, United States). Data were normalized to baseline and used for further analyses. All the parameters were maintained constant for an individual recorded neuron in treatments of ACSF, drugs, and recovery. Values are expressed as the mean ± SEM. One-way and repeated-measures analysis of variance (ANOVA) followed by Tukey *post hoc* test or two-way ANOVA (SPSS Software, Chicago, IL, United States) was used to determine the level of statistical significance between groups of data. *P* < 0.05 was considered to indicate a statistically significant difference between experimental groups.

## Results

### Effect of CRF on Facial Stimulation–Induced MLI-PC Synaptic Transmission in Cerebellar Cortex

**FIGURE 2:**
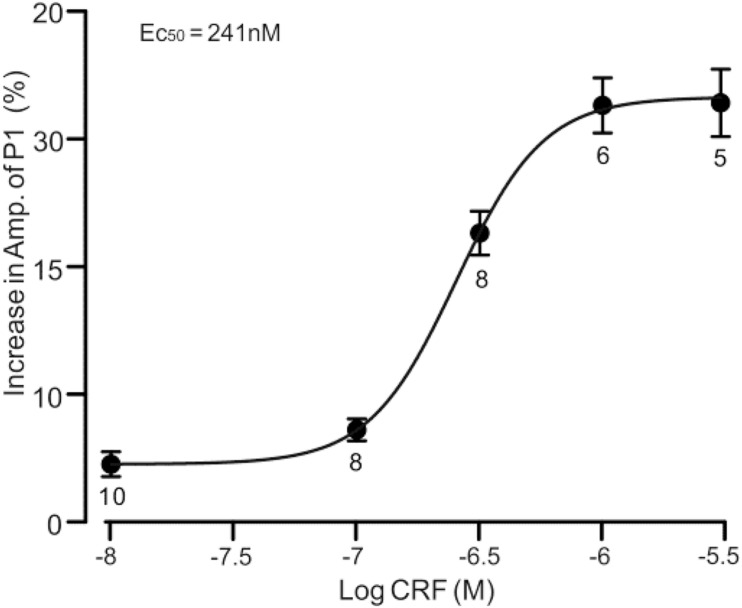
A concentration–response curve shows the CRF-induced increase in amplitude of P1. The EC_50_ value obtained from the curve was 241 nM. Error bars indicate SEM.

Under cell-attached recording conditions, a total of 70 cells were identified as cerebellar PCs by exhibiting SS and CS activity (Figure 1A, asterisk). Consistent with our previous studies (Chu et al., 2011; Bing et al., 2015), air-puff stimulation of ipsilateral whisker pad (10 ms; 60 psi) evoked a sequence of excitatory component (N1) and an inhibitory component (P1) followed by a pause of SS firing (Figures 1A,B); P1 was identified as MLI-PC GABAergic synaptic transmission onto cerebellar PCs, whereas N1 was induced by parallel fiber volley (Chu et al., 2011, 2012; Supplementary Material). Molecular layer microapplication of CRF (300 nM) had no significant effect on the frequency of spontaneous SS firing (*P* = 0.46; *n* = 8 cells in eight mice; Figures 1B,G), but induced a time-dependent increase in amplitude of P1 (Figure 1C). The normalized amplitude of P1 was 119.5% ± 6.8% of baseline (100% ± 5.6%; *P* < 0.001; *n* = 8 cells in eight mice; Figure 1D). CRF also induced a significant increase in the facial stimulation–evoked SS pause to 132.3% ± 6.3% of baseline (100% ± 5.3%; *P* < 0.001; *n* = 8; Figures 1A,E). However, application of CRF did not change the amplitude of N1; the normalized amplitude of N1 was 103.1% ± 5.4% of baseline (100.0% ± 2.2%; *P* = 0.68, *n* = 8 cells in eight mice, Figure 1F). The CRF-induced increase in amplitude of P1 was concentration dependent (Figure 2), with a 50% effective concentration (EC_50_) of 241 nM. The concentration of 1 μM CRF induced an increase in the amplitude of P1 to 32.6% ± 2.2% of baseline (*P* < 0.001 vs. baseline; *n* = 6 cells in six mice). These results indicate that molecular layer application of CRF induces a dose-dependent increase in the facial stimulation–evoked MLI-PC synaptic transmission but without change in parallel fiber excitatory inputs *in vivo* in mice.

**FIGURE 3:**
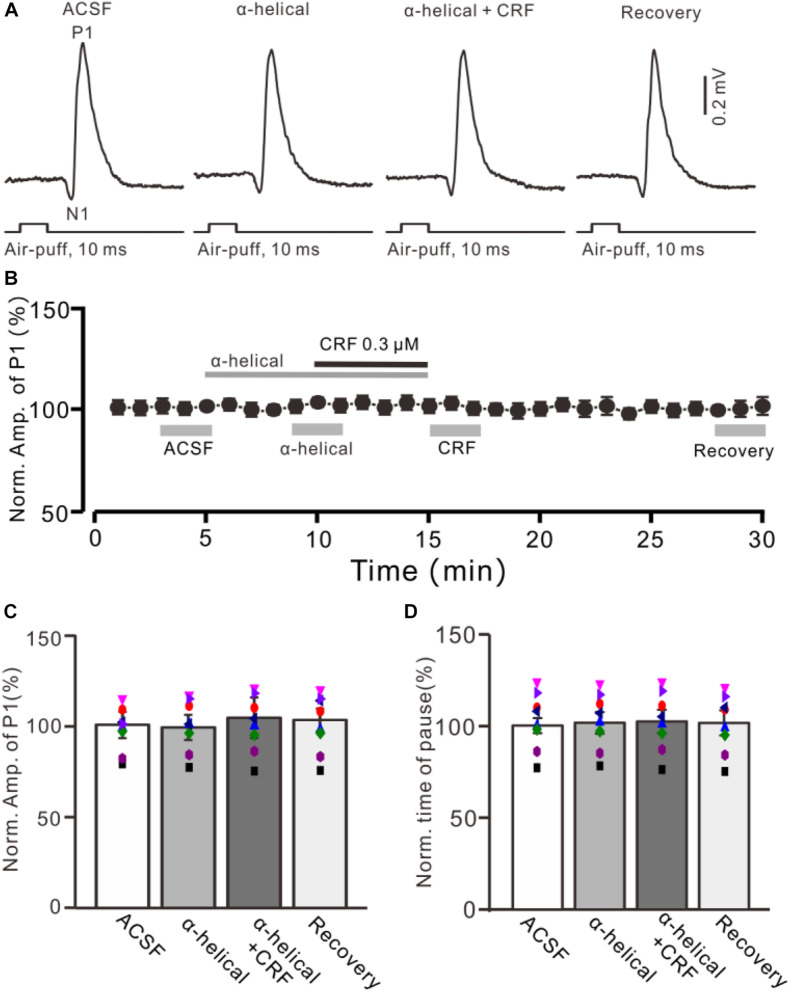
A non-selective CRF receptors antagonist abolished the effect of CRF on the facial stimulation–evoked MLI-PC synaptic transmission. **(A)** Cell-attached recording traces (average of five consecutive recordings in each trace) showing air-puff stimulation (10 ms, 60 psi)–evoked responses in a cerebellar PC during application of ACSF, α-helical (1 μM), α-helical (1 μM) + CRF (0.3 μM), and recovery. **(B)** Summary of data (*n* = 8) shows the time course of normalized amplitude of P1. **(C)** Bar graph with individual data (*n* = 8) shows the normalized amplitude of P1 in the treatments with ACSF, α-helical, α-helical + CRF (0.3 μM), and recovery [indicated by gray bars in panel **(B)**]. **(D)** Pooled data showing the normalized pause of simple spike firing during application of ACSF, α-helical, α-helical + CRF (0.3 μM), and recovery.

**FIGURE 4:**
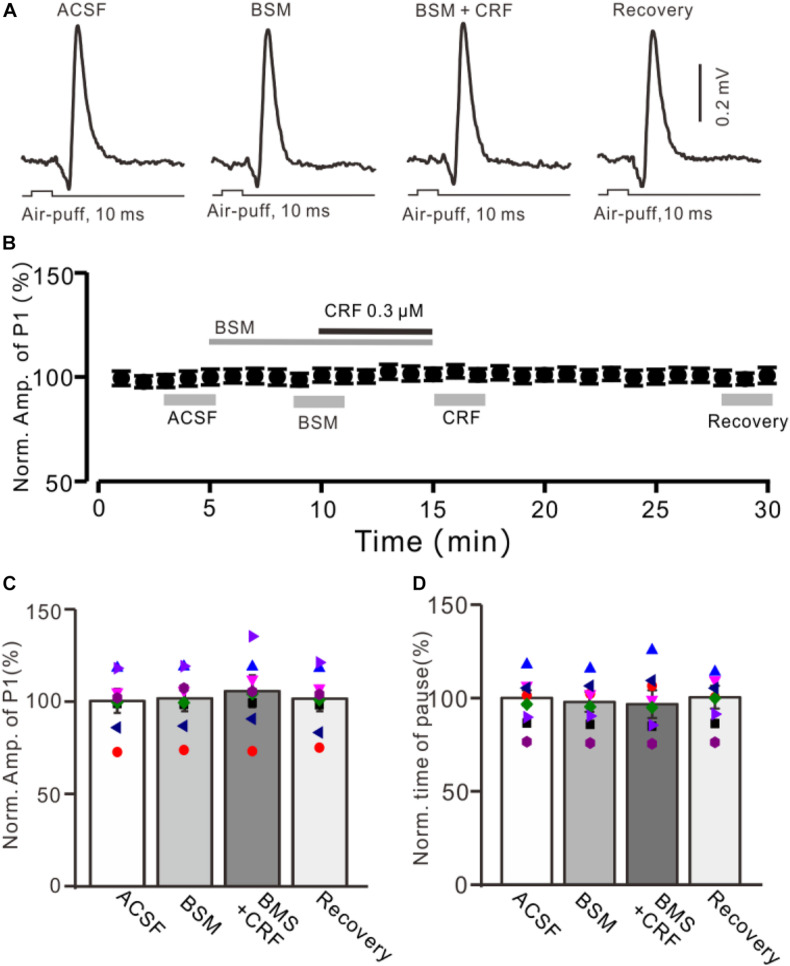
CRF-induced enhancement of MLI-PC synaptic transmission was prevented by CRF-R1 selective antagonist. **(A)** Cell-attached recording traces (average of five consecutive recordings in each trace) showing air-puff stimulation (10 ms, 60 psi)–evoked responses in a cerebellar PC during application of ACSF, BMS-763534 (BMS, 0.1 μM), and BMS + CRF (0.3 μM). **(B)** Summary of data (*n* = 8) showing the time course of normalized amplitude of P1 in each treatment. **(C)** Bar graph with individual data (*n* = 8) showing the normalized amplitude of P1 during application of ACSF, BMS-763534 (BMS), BMS + CRF, and washout of CRF (recovery) [indicated by gray bars in panel **(C)**]. **(D)** Pooled data showing the normalized pause of SS in each treatment.

**FIGURE 5:**
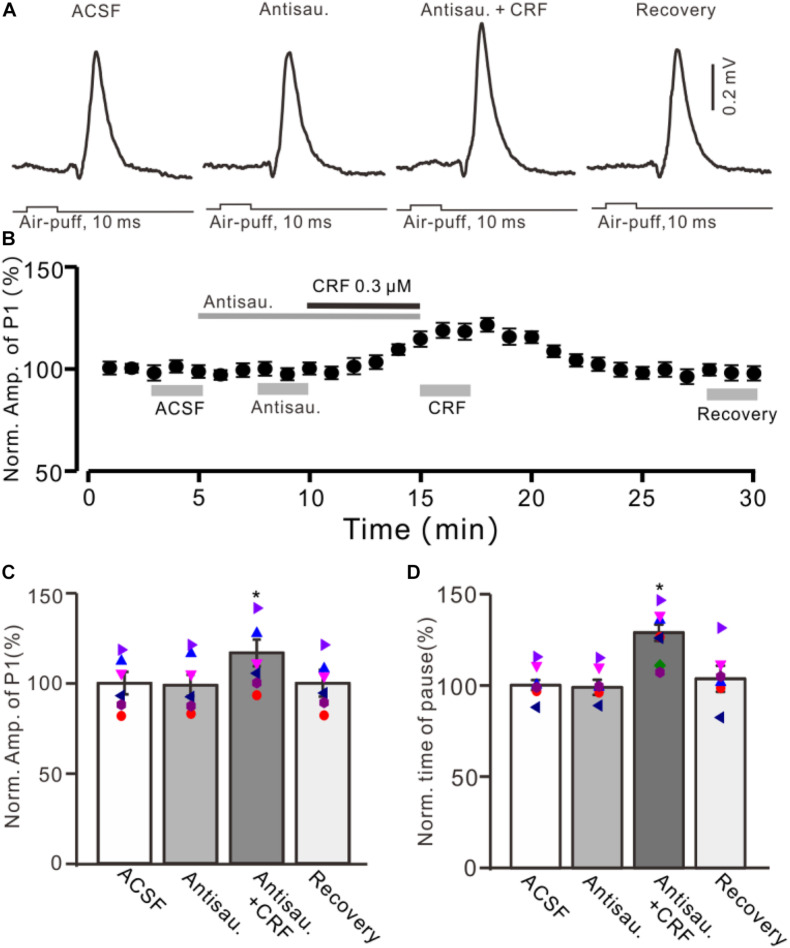
Blocking CRF-R2 failed to prevent the CRF-induced enhancement of MLI-PC synaptic transmission. **(A)** Cell-attached recording traces (average of five consecutive recordings in each trace) showing air-puff stimulation (10 ms, 60 psi)–evoked responses in a cerebellar PC during application of ACSF, antisauvagine-30 (0.2 μM), antisauvagine-30 (0.2 μM) + CRF (0.3 μM), and washout of CRF (recovery). **(B)** Summary of data (*n* = 6) showing the time course of normalized amplitude of P1. **(C)** Bar graph with individual data showing the normalized amplitude of P1 during application of ACSF, antisauvagine-30, antisauvagine-30 + CRF, and washout of CRF (recovery) [indicated by gray bars in panel **(C)**]. **(D)** Pooled data showing the normalized pause of simple spike firing during application of ACSF, antisauvagine-30, antisauvagine-30 + CRF, and washout of CRF (recovery). *P < 0.05 vs. ACSF.

### CRF Enhances MLI-PC Synaptic Transmission via Activation of CRF-R1

Bath application of a non-selective CRF receptor antagonist, α-helical CRF-(9-14) (1 μM), for 5 min did not significantly change the amplitude of P1 (Figures 3A,B); the normalized amplitude was 101.3% ± 6.3% of control (100.0% ± 6.8%; *P* = 0.74; *n* = 8 cells in eight mice; Figure 3C). In the presence of α-helical CRF-(9-14), microapplication of CRF failed to increase amplitude of P1, with the normalized amplitude of P1 being 103.8% ± 11.1% of baseline (*P* = 0.65; *n* = 8 cells in eight mice; Figure 3C). In addition, application of α-helical CRF-(9-14) did less effect on the facial stimulation–evoked SS pause but prevented the CRF-induced increase in the SS pause, with the normalized SS pause being 102.2% ± 6.4% of baseline (*P* = 0.68; *n* = 8 cells in eight mice; Figure 3D).

We further employed a selective CRF-R1 antagonist, BMS-763534 (BMS, 200 nM), to examine whether CRF induced increase in the amplitude of P1 via CRF-R1. Bath administration of BMS for 200 s did not significantly change amplitude of P1, with a normalized amplitude of 101.4% ± 7.0% of control (100.0% ± 6.5%; *P* = 0.76; *n* = 8 cells in eight mice; Figures 4A,B). In the presence of BMS-763534, microapplication of CRF failed to increase amplitude of P1 (normalized amplitude of P1 was 105.2% ± 8.6% of control; *P* = 0.54; *n* = 8 cells in eight mice; Figure 4C). In addition, BMS did not affect the facial stimulation–evoked SS pause and the CRF-induced increase in the SS pause (Figure 3D).

Moreover, we used a selective CRF-R2 antagonist, antisauvagine-30 (200 nM), to determine whether the CRF-induced increase in amplitude of P1 was involved in CRF-R2. As shown in Figure 5, bath administration of antisauvagine-30 for 200 s did not significantly change the amplitude of P1 (normalized amplitude of P1 was 97.6% ± 6.3% of control; 100.0% ± 5.7%; *P* = 0.62; *n* = 6 cells in six mice; Figures 5A–C). In the presence of antisauvagine-30, microapplication of CRF still induced an increase in amplitude of P1, with a normalized SS firing rate of 113.6% ± 5.9% of control (*P* = 0.026; *n* = 6 cells in six mice; Figures 5B,C). In addition, antisauvagine-30 did not significantly change the facial stimulation–evoked SS pause and failed to prevent the CRF-induced increase in the SS pause, with the normalized SS pause being 128.6% ± 4.4% of baseline (*P* < 0.001; *n* = 6 cells in six mice; Figure 5D).

### CRF Enhances MLIs Excitability via CRF-R1

**FIGURE 6:**
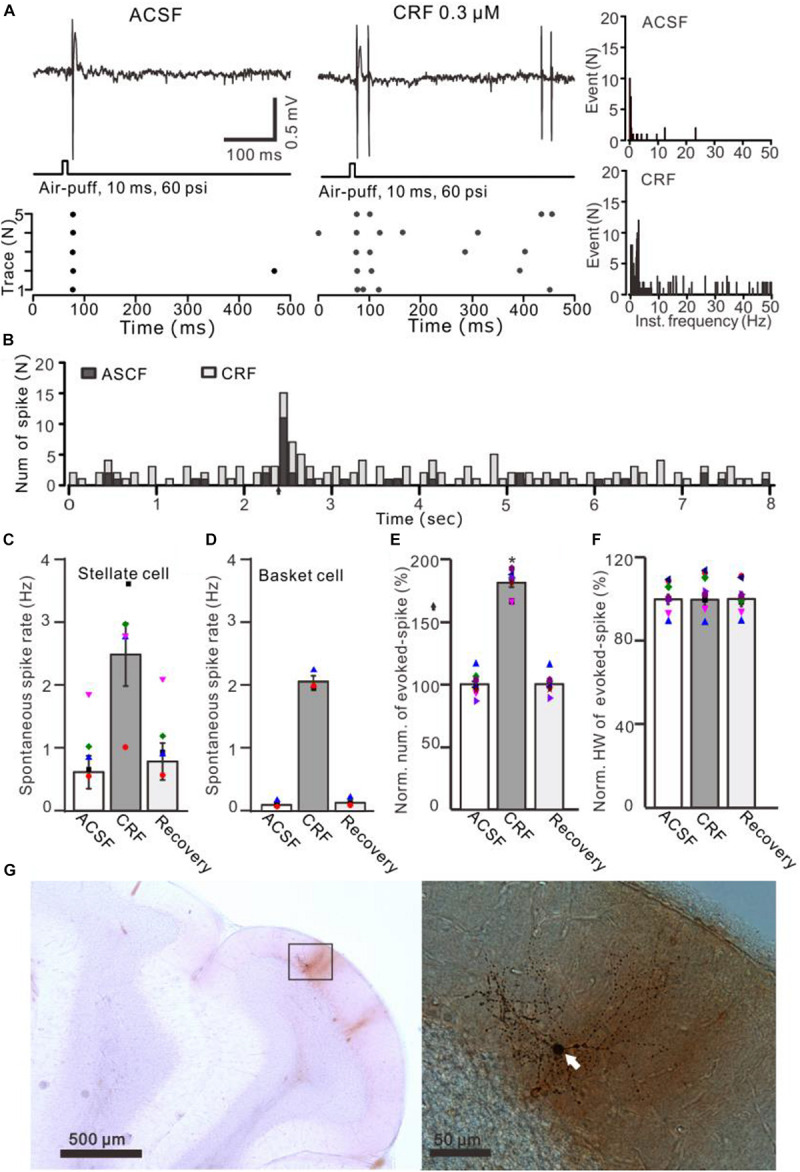
Effects of CRF on the spontaneous and the facial stimulation–evoked spike firing activity of MLIs. **(A)** Upper: Representative traces showing the spontaneous and the facial stimulation–evoked spike firing of an MLI during application of ACSF, 0.3 μM CRF, and recovery (wash). Lower: Raster plot of the spike events of the MLI before and after application of CRF. **(B)** Perievent histograms of an MLI [shown in panel **(A)**] discharge in the presence of ACSF (black) and CRF (gray) (arrow denotes air-puff stimulation). **(C)** Bar graph showing the effect of CRF on spontaneous spike firing rate of stellate-type MLIs (*n* = 5). **(D)** Bar graph showing the effect of CRF on spontaneous spike firing rate of basket-type MLIs (*n* = 3). **(E,F)** Bar graphs with individual data show the normalized number **(E)** half-width [HW; **(F)**] of the facial stimulation–evoked spike firing in each treatment (*n* = 8). **(G)** Photomicrographs showing the properties of the recorded-MLI filled with neurobiotin by juxtacellular stimulation, which identified as basket-type MLI (arrow). *P < 0.05 vs. ACSF.

**FIGURE 7:**
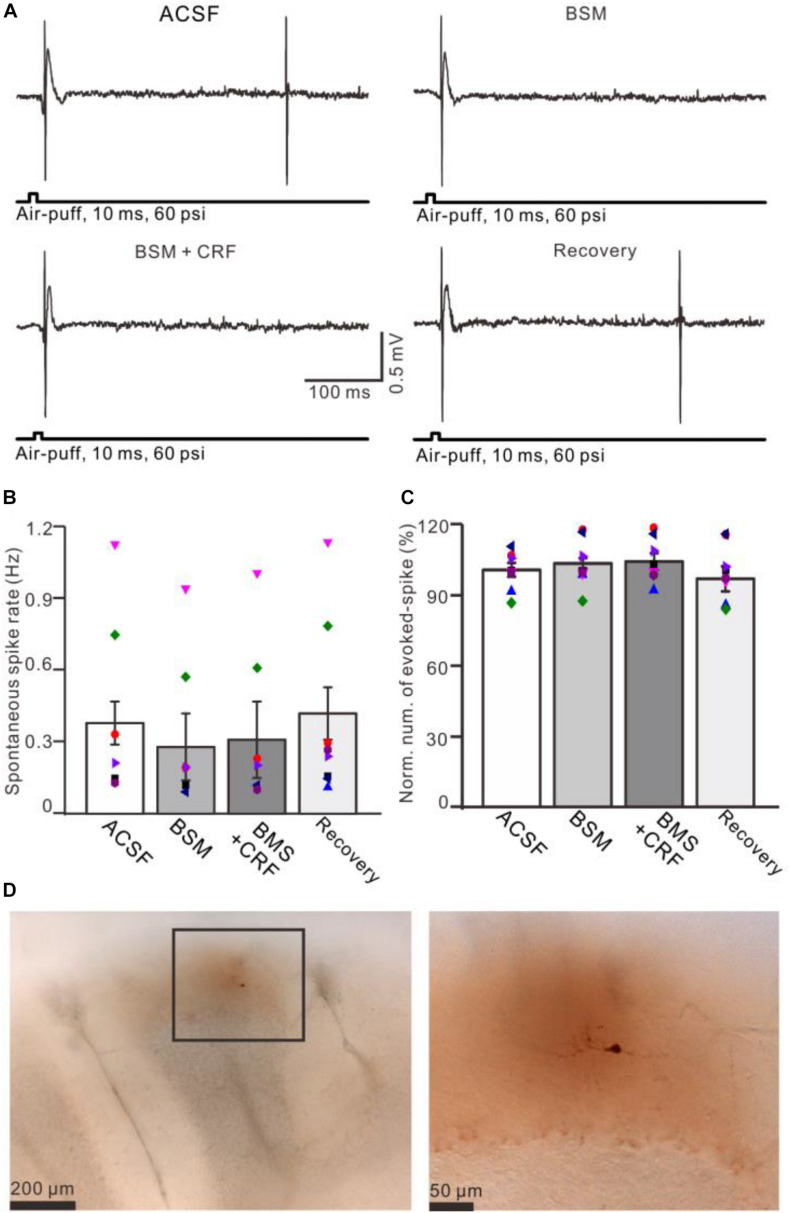
Blockade of CRF-R1 prevented the effect of CRF on spike firing activity of MLIs. **(A)** Representative traces showing the spontaneous and the facial stimulation–evoked spike firing of an MLI during application of ACSF, BMS-763534 (BMS, 0.1 μM), BMS + CRF (0.3 μM), and recovery. **(B)** Bar graph with individual data shows the normalized number of the facial stimulation–evoked spike firing in each treatment (*n* = 8). **(C)** Pooled data (*n* = 8) showing the effect of CRF on spontaneous spike firing rate (*n* = 8). **(D)** Photomicrographs showing the MLI filled with neurobiotin, which is identified as stellate-type MLI.

Because MLIs are inhibitory neurons of PCs that express CRF-R1 (Ito, 1984; Tian et al., 2008; Tao et al., 2009), the CRF enhances MLI-PC synaptic transmission through parallel fiber-MLI excitatory inputs. We examined the effect of CRF on the facial stimulation–evoked spike firing activity of MLIs by cell-attached recordings accompanied with neurobiotin juxtacellular labeling technique (Holtzman et al., 2006; Liu et al., 2014). A total of 16 neurons were identified as MLIs by their location in the molecular layer, spontaneous spike firing properties and confirmed by neurobiotin histochemistry (Figure 6), which consists of 12 stellate-type and 4 basket-type identified by the presence of characteristic terminals and their dendritic trees (Holtzman et al., 2006; Chu et al., 2012; Liu et al., 2014). The basket-type MLIs processed somas with a mean diameter is 12.22 ± 0.37 μm (*n* = 4 cells). Their identification depends on the presence of characteristic terminals that dropped descending collaterals to wrap around several somas of PCs (Figure 6G). The stellate-type MLIs possessed somas with a mean diameter of 9.31 ± 0.18 μm (*n* = 12), which were identified by location on molecular layer and short dendrites (Figure 7D). Within these MLIs, the facial stimulation evoked spike firing in three basket-type MLIs and five stellate-type MLIs.

**FIGURE 8:**
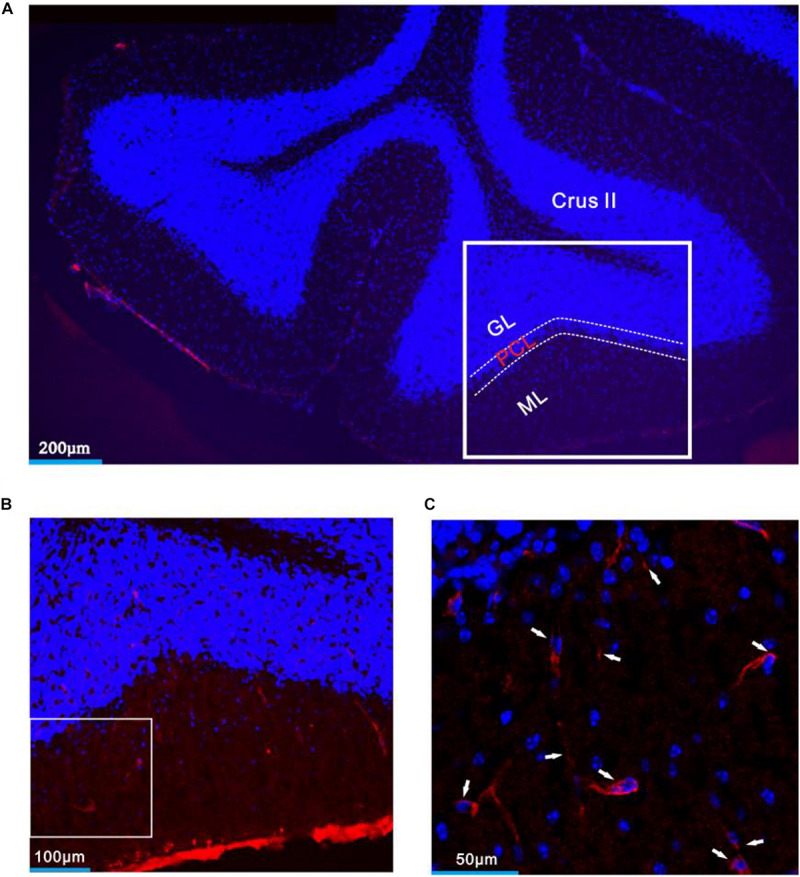
CRF-R1 was expressed in MLIs of mouse cerebellar Crus II. **(A)** A digital micrograph that shows the confocal image of DAPI (blue) in cerebellar lobule Crus II. DAPI is a blue nucleic acid dye that preferentially dyes the dsDNA of cells. **(B)** Higher magnifications of the boxed area in panel **(A)**. **(C)** Higher magnification of the boxed area in panel **(B)** shows CRF-R1 immunoreactivity expressed on MLIs (red; arrows). ML, molecular layer; PCL, Purkinje cell layer; GL, granular layer.

Application of CRF (300 nM) increased spike firing rate of both basket-type MLIs and stellate-type MLIs (Figures 6A,B). In the presence of CRF, the spontaneous spike firing rate of basket-type MLIs was increased from 0.1 ± 0.03 Hz to 2.05 ± 0.0.09 Hz (*P* = 0.026; *n* = 3 in three mice; Figure 6C), and the spontaneous spike firing rate of stellate-type MLIs was increased from 0.61 ± 0.26 Hz to 2.48 ± 0.5 Hz (*P* = 0.014, *n* = 5 in three mice; Figure 6D). Notably, CRF induced a significant increase in the number of the facial stimulation–evoked action potentials; the normalized number of the evoked-action potential was 181.2% ± 3.7% of baseline (*P* < 0.001; *n* = 8 cells in eight mice; Figure 6E). However, CRF did not significant change half-width of the evoked-action potential; the normalized value of half-width was 102.8% ± 2.6% of baseline (*P* = 0.76; *n* = 8 in eight mice; Figure 6F). Furthermore, bath application of CRF-R1 antagonist, BMS-763534, for 200 s did not significantly change the spontaneous spike firing rate, as well the facial stimulation–evoked action potentials. The frequency of spontaneous spike firing was 0.28 ± 0.14 Hz, which was similar to that in ACSF (0.38 ± 0.09 Hz; *P* = 0.47; *n* = 8; Figures 7A,B), and the normalized number of the evoked action potentials was 102.8% ± 2.2% of control (100.0% ± 2.9%; *P* = 0.76; *n* = 8 cells in eight mice; Figures 7A,C). In the presence of BMS-763534, microapplication of CRF (300 nM) failed to increase the spontaneous spike firing rate and the evoked action potentials. The frequency of spontaneous spike firing was 0.31 ± 0.16 Hz, which was similar to that in ACSF (0.38 ± 0.09 Hz; *P* = 0.56; *n* = 8; Figures 7A,B), and the normalized number of the evoked action potentials was 103.6% ± 3.5% of control (100.0% ± 2.9%; *P* = 0.67; *n* = 8 cells in eight mice; Figures 7A,C). Moreover, CRF-R1 immunoreactivity was found on somas of MLIs (Figure 8). These results indicate that CRF induced increases in spontaneous spike firing rate and the number of facial stimulation–evoked action potential in MLIs via CRF-R1, suggesting that CRF acts on CRF-R1 and enhances the excitability of MLIs, resulting in an increase in the facial stimulation–evoked MLI-PC synaptic transmission *in vivo* in mice.

## Discussion

In this study, we investigated the effect of CRF on the facial stimulation–evoked cerebellar cortical MLI-PC synaptic transmission in urethane-anesthetized mice by *in vivo* cell-attached recording, neurobiotin juxtacellular labeling techniques, and pharmacological methods. The results showed that microapplication of CRF in cerebellar molecular layer induced a concentration-dependent increase in amplitude of the facial stimulation–evoked MLI-PC synaptic transmission accompanied with an increase in pause of SS firing, which was abolished by either a non-selective CRF receptor antagonist, α-helical CRF-(9-14), or a selective CRF-R1 antagonist, BMS-763534. Moreover, application CRF not only induced a significant increase in spontaneous spike firing rate, but also produced a significant increase in the number of the facial stimulation–evoked action potential in MLIs. The effect of CRF on the activity of MLIs was abolished by blockade of CRF-R1 with BMS-763534. These results indicate that CRF increases excitability of MLIs, resulting in an enhancement of the facial stimulation–evoked MLI-PC synaptic transmission via CRF-R1 *in vivo* in mice.

### CRF Modulates MLI-PC Synaptic Transmission by Enhancing Cerebellar MLIs Activity

The cerebellar cortical MLIs include basket cells and stellate cells, which receive excitatory input from parallel fibers and inhibitory input from other interneurons (Palay and Chan-Palay, 1974; Llano and Gerschenfeld, 1993; Häusser and Clarck, 1997; Mittmann et al., 2005). The basket-type MLIs inhibit the somas of PC, whereas the stellate-type MLIs innervate the dendrites of PCs (Palay and Chan-Palay, 1974; Huang et al., 2007). The sensory information transferred to cerebellar cortex through climbing fiber and MF-GC-PF pathways, which induces synaptic transmission (Ito, 1984). Our previous studies showed that the facial stimulation evoked excitation of MLIs resulting in an inhibition of PCs under *in vivo* conditions, suggesting that the sensory stimulation–evoked MLI-PC synaptic transmission and plasticity play a critical role on controlling the spike firing of PCs (Chu et al., 2011, 2012; Bing et al., 2015; Ma et al., 2019). It has been demonstrated that acute stress not only disrupts sensory information processing in the central nervous system, but also impairs motor coordination and a variety of cognitive processes such as sustained attention and working memory (Clark et al., 1986; Grillon and Davis, 1997; Ermutlu et al., 2005). A previous study demonstrated that administration of CRF in locus coeruleus induced a dose-dependent suppression of sensory-evoked discharge in ventral posterior medial thalamic and barrel field cortical neurons (Devilbiss et al., 2012). The present results showed that molecular layer microapplication CRF induced dose-dependently facilitation of the facial stimulation–evoked MLI-PC synaptic transmission but without effect parallel fiber volley, indicating that CRF modulates MLI-PC synapse activity without change in parallel fiber excitatory inputs under *in vivo* in mice. Importantly, molecular layer microapplication of CRF induced increases in spontaneous spike firing rate and the number of facial stimulation evoked action potential. These results suggest that CRF increases excitation of MLIs, resulting in an enhancement of the facial stimulation–evoked MLI-PC synaptic transmission.

### CRF Modulates the Facial Stimulation–Evoked MLI-PC Synaptic Transmission Through CRF-R1

Both CRF-R1 and CRF-R2 have been found in the adult rodent cerebellum (Bishop, 1990; Bishop et al., 2000; Lee et al., 2004). CRF-R1 is expressed throughout all lobules of the cerebellar cortex, including the primary dendrites and somas of PCs, MLIs and granular cells (Tian et al., 2008; Tao et al., 2009; Refojo et al., 2011; K``uhne et al., 2012), and CRF has been found to modulate neuronal spontaneous spike firing activity in cerebellar cortex via CRF receptors (Fox and Gruol, 1993; Bishop et al., 2006; Tao et al., 2009; Libster et al., 2015; Gunn et al., 2017; Prouty et al., 2017; Wang et al., 2018). Our previous results showed that cerebellar molecular layer application of CRF increased excitation of PCs through CRF-R2 at presynaptic sites *in vivo* in mice (Wang et al., 2018). In addition, it has been demonstrated that CRF selectively excites glutamatergic neurons rather than GABAergic neurons in the cerebellar interpositus nucleus through both CRF-R1 and CRF-R2, by activation of inward rectifier K^+^ channel and/or hyperpolarization-activated cyclic nucleotide-gated channel (Wang et al., 2017). In this study, we found that the effect of CRF on MLI-PC synaptic transmission was abolished by a non-selective CRF receptor antagonist, α-helical CRF-(9-14), indicating that CRF increased the MLI-PC synaptic transmission via CRF receptors. Furthermore, a selective CRF-R1 antagonist, BMS-763534 completely prevented the effect of CRF on MLI-PC synaptic transmission, indicating that CRF enhances the MLI-PC synaptic transmission via CRF-R1. Moreover, a selective CRF-R2 antagonist failed to block the effect of CRF on MLI-PC synaptic transmission, confirming that CRF increasing the MLI-PC synaptic transmission is not dependent on CRF-R2.

CRF-regulated neurotransmitter release through CRF-R1 has been demonstrated previously (Gunn et al., 2017; Varodayan et al., 2017). First, CRF primarily acted at presynaptic CRF-R1 to produce opposite effects on the central nucleus of the amygdala glutamate release and modulated the glutamatergic synapses (Varodayan et al., 2017). Furthermore, blockade endogenous CRF-R1 depressed the spontaneous excitatory transmission onto CA3 pyramidal cells, indicating that endogenous CRF modulated hippocampal network and memory via CRF-R1 (Gunn et al., 2017). Moreover, CRF-R1 has critical roles in regulating particular forms of cerebellar learning both at the cellular and behavioral levels, but without an effect on baseline motor skills (Ezra-Nevo et al., 2018a). The present results are consistent with previous studies (Refojo et al., 2011; K``uhne et al., 2012; Ezra-Nevo et al., 2018a), suggesting that CRF modulates MLI-PC synaptic transmission via CRF-R1. Importantly, application of CRF-R1 antagonist, BMS-763534, did not significantly change the spontaneous spike firing rate and the facial stimulation–evoked action potentials, but completely prevented the CRF-induced increases in the spontaneous spike firing rate and the evoked action potentials of MLIs. These results indicate CRF-induced increases in spontaneous spike firing rate and the number of facial stimulation–evoked action potential in MLIs via CRF-R1, suggesting that CRF acts on CRF-R1 and enhances the excitability of MLIs, resulting in an increase in the facial stimulation–evoked MLI-PC synaptic transmission *in vivo* in mice.

## Data Availability Statement

The raw data supporting the conclusions of this article will be made available by the authors, without undue reservation.

## Ethics Statement

The animal study was reviewed and approved by the experimental procedures were approved by the Animal Care and Use Committee of Yanbian University and were in accordance with the animal welfare guidelines of the United States National Institutes of Health. The permit number is SYXK (Ji) 2011-006.

## Author Contributions

D-LQ, W-YW, Y-ZL, and M-CW conceived and designed the experiments. W-YW, M-CW, YL, and H-WW performed the experiments. C-PC and D-LQ analyzed the data. H-WW contributed reagents, materials, and analysis tools. C-PC, D-LQ, and HJ wrote the manuscript. All authors contributed to the article and approved the submitted version.

## Conflict of Interest

The authors declare that the research was conducted in the absence of any commercial or financial relationships that could be construed as a potential conflict of interest.
