# Supplementary material for: Corticotrophin-Releasing Factor Modulates the Facial Stimulation-Evoked Molecular Layer Interneuron-Purkinje Cell Synaptic Transmission in vivo in Mice
Source: Front Cell Neurosci. 2020 Nov 26;14:563428. doi: 10.3389/fncel.2020.563428 (PMC7726213; doi:10.3389/fncel.2020.563428)
Supplement: Supplementary file 1 [file Data_Sheet_1.PDF]

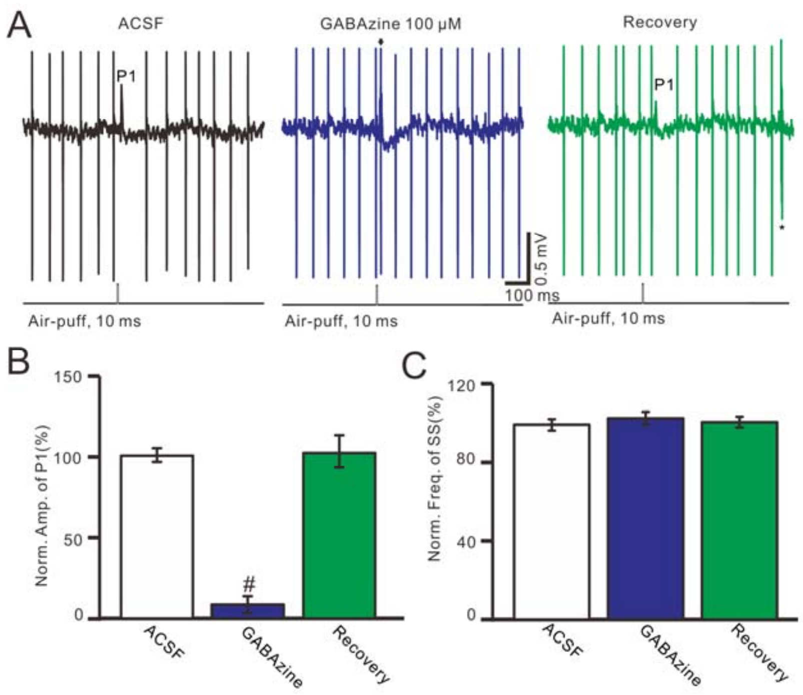

Identification of the facial stimulation-evoked MLI-PC synaptic transmission in vivo in mice. (A) Representative cell-attached recording traces showing air-puff stimulation (10 ms, 60 psi; arrows)-evoked responses in a cerebellar PC during application of ACSF, GABAzine (100 μM) and recovery (washout). (B) Summary of data (n = 6) show the normalized amplitude of P1 during application of ACSF, GABAzine and recovery. (C) Summary of data (n = 6) show the normalized spontaneous simple spike firing rate during application of ACSF, GABAzine and recovery.
